# Trends in Diagnosing Obesity: The CALOR Study

**DOI:** 10.1002/oby.70264

**Published:** 2026-07-18

**Authors:** Deborah B. Horn, Eva Lesén, Christen M. Gray, Magnus Bjursell, Hironori Waki

**Affiliations:** ^1^ Center for Obesity Medicine and Metabolic Performance Departments of Medicine and Surgery, UT McGovern Medical School Houston Texas USA; ^2^ CVRM Evidence Strategy, BioPharmaceuticals Medical, AstraZeneca Gothenburg Sweden; ^3^ Real World Data Science, BioPharmaceuticals Medical, AstraZeneca Cambridge UK; ^4^ CVRM Medical, BioPharmaceuticals Medical, AstraZeneca Gothenburg Sweden; ^5^ Department of Metabolism and Endocrinology Akita University Graduate School of Medicine Akita Japan

**Keywords:** burden, comorbidity, diagnosis, interrelated disease, obesity

## Abstract

**Objective:**

This study aimed to describe characteristics of people with elevated BMI by presence versus absence of obesity diagnosis and trends over time.

**Methods:**

Data from adults with recorded BMI ≥ 30 kg/m^2^ (United States [US]; Optum's deidentified Clinformatics Data Mart database) or ≥ 25 kg/m^2^ (Japan; Medical Data Vision) between 2016 and 2024 were included. Characteristics were described at index (most recent BMI or obesity diagnosis), stratified by presence versus absence of prior recorded obesity diagnosis.

**Results:**

In the United States (*N* = 3,584,120; 70.1% with obesity diagnosis), prevalence of interrelated diseases was high overall and higher in those with versus without obesity diagnosis, e.g., type 2 diabetes (49.3% vs. 29.9%), chronic kidney disease (32.9% vs. 18.8%), and heart failure (23.3% vs. 9.8%). In Japan (*N* = 3,140,900; 2.3% with obesity diagnosis), interrelated disease burden was also higher in those with versus without obesity diagnosis, e.g., type 2 diabetes (42.2% vs. 25.9%) and chronic kidney disease (14.3% vs. 8.6%). In the United States, interrelated disease burden was generally higher for those first diagnosed in 2024 versus those diagnosed in 2016–2020.

**Conclusions:**

In people with elevated BMI in the United States and Japan, there was underdiagnosis of obesity and a high prevalence of interrelated diseases.

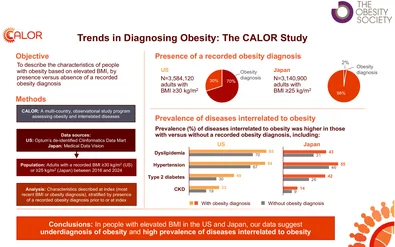

## Study Importance

- What is already known?
  ○ Obesity is a complex, multifactorial disease that is often undiagnosed in clinical practice.
  ○ Considering the recent developments in obesity disease awareness, diagnosis criteria, and treatment options, there is a need to evaluate contemporary patterns in obesity diagnosis and the burden of diseases interrelated to obesity.
- What does this study add?
  ○ In people with elevated BMI in the United States (BMI ≥ 30 kg/m^2^) and Japan (≥ 25 kg/m^2^), 70.1% and 2.3% of individuals, respectively, had a recorded obesity diagnosis.
  ○ The burden of diseases interrelated to obesity was higher in those with versus without a recorded obesity diagnosis and, in the United States, was generally higher for those first diagnosed more recently.
- How might these results change the direction of research or the focus of clinical practice?
  ○ Highlighting that obesity is a disease and improving obesity diagnosis rates represent a significant clinical opportunity and can improve access to healthcare for obesity and interrelated diseases.
  ○ Understanding the burden of obesity may encourage healthcare systems to prioritize diagnosis and better management of obesity and its interrelated diseases.

## Graphical abstract

Data from the CALOR study suggest that in people with elevated BMI in the US and Japan, there is underdiagnosis of obesity and a high prevalence of diseases interrelated to obesity.

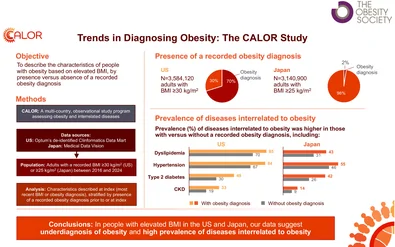

## 1 Introduction

Obesity is a complex, multifactorial disease characterized by abnormal or excessive accumulation of body fat [1]. The prevalence of obesity has increased steadily over the past few decades, reaching epidemic levels [1, 2], and is now one of the most significant public health challenges [3]. However, it is often undiagnosed in clinical practice [4, 5, 6, 7, 8, 9, 10].

Obesity can be diagnosed using body mass index (BMI) [11]—a widely used surrogate marker of adiposity [12]—among other methods. However, BMI may misclassify adiposity at the individual level (under‐ or over estimating excess adiposity), and contemporary frameworks have highlighted the value of complementing BMI with additional assessments of adiposity and health impact [13, 14]. Prior research indicates that receiving an obesity diagnosis is associated with improved treatment guideline adherence and improved health outcomes for people with obesity [4, 5, 7, 10]. Specifically, people with an obesity diagnosis were more likely to be offered an obesity intervention and management plan [4, 7, 10]. Additionally, an obesity diagnosis was an independent predictor of loss of ≥ 5% total body weight over 9–15 months [5].

The standard of care for obesity includes lifestyle intervention, pharmacotherapy, and bariatric surgery [11]. Recently, glucagon‐like peptide‐1 receptor agonists (GLP‐1RAs) have emerged as a key component of medical therapy, as they promote significant weight loss and improve cardiovascular, renal, and metabolic (CVRM) complications [15, 16]. Furthermore, treatment continues to evolve, with multiple agents either available or in development, including dual or triple receptor agonists targeting the glucagon‐like peptide‐1 (GLP‐1) receptor, glucose‐dependent insulinotropic polypeptide receptor, and glucagon receptor [17] and amylin agonists as monotherapy or combined with GLP‐1RAs [18, 19].

In addition to treatment, the landscape of obesity disease awareness and diagnosis criteria continues to expand and evolve [13, 20, 21]. Importantly, there has been a paradigm shift toward recognizing obesity as a complex chronic disease associated with multiple interrelated diseases and acknowledging that it requires compassionate, comprehensive, and patient‐centered care that is free from stigma and bias [21, 22]. Given these advances in the understanding of obesity and its treatment, there is a need to evaluate contemporary developments in obesity diagnosis patterns and the burden of diseases interrelated to obesity.

CALOR is a multicountry, observational study program assessing the characteristics, occurrence, and risks of diseases interrelated to obesity. Here, we describe (1) the contemporary characteristics of people with obesity based on elevated BMI, with versus without a recorded diagnosis of obesity, and (2) the trends in diagnosing obesity over time. Analyses were performed across two geographically diverse countries with different healthcare systems and ethnicity‐related patterns of pathophysiology—the United States (US) and Japan [23, 24].

## 2 Methods

### 2.1 Design and Data Sources

**FIGURE 1:**
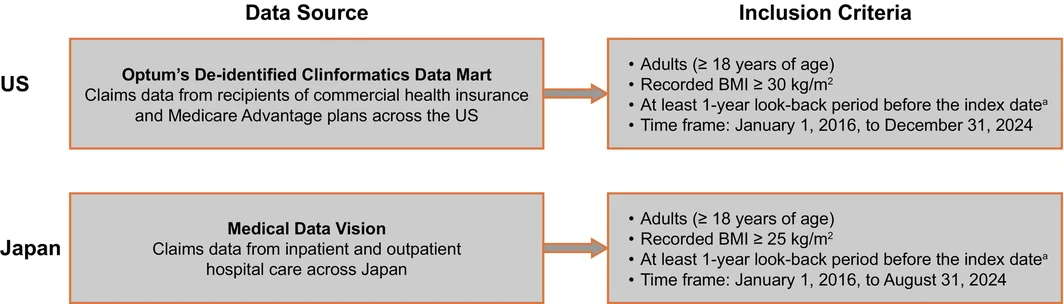
CALOR study design. ^a^Index date: most recent record of BMI ≥ 25 mg/kg^2^ (Japan) or ≥ 30 mg/kg^2^ (United States) or an obesity diagnosis record. [Color figure can be viewed at wileyonlinelibrary.com]

The CALOR study program is based on longitudinal data collected retrospectively from healthcare claims and electronic medical records. The current analyses are based on data from the United States and Japan (Figure 1).

Data from the United States were obtained from Optum's deidentified Clinformatics Data Mart database, an administrative health database comprising claims data from recipients of commercial health insurance and Medicare Advantage plans across the United States. The database includes detailed enrollment information, diagnoses, and procedures recorded in inpatient and outpatient care settings, prescription medications, and some laboratory results. Data from Japan were obtained from Medical Data Vision, a database built out of claims from hospitals in Japan. Data are collected directly from hospitals all over Japan, regardless of insurance status. The database includes information on diagnoses and procedures recorded in inpatient and outpatient hospital care, administration of medications in hospitals, and prescriptions issued from the hospital setting; laboratory test results were available from a subset of hospitals [25].

The US data source included deidentified data in compliance with the Health Insurance Portability and Accountability Act requirements; neither informed consent nor institutional review board approvals were required. According to the Japanese Ethical Guidelines for Medical and Health Research Involving Human Subjects, ethical approval and informed consent do not apply to the use of deidentified secondary data.

### 2.2 Study Population

Participants were adults (≥ 18 years of age) who had a recorded BMI that met the threshold for obesity in their country, ≥ 30 kg/m^2^ in the United States and ≥ 25 kg/m^2^ in Japan [24], between January 1, 2016, and December 31, 2024 (United States) and between January 1, 2016, and August 31, 2024 (Japan).

Participants' index date was defined as the most recent record of BMI ≥ 30/25 kg/m^2^ (US/Japan) or a recorded diagnosis of obesity. All participants were required to have at least a 1‐year look‐back period before the index date, while all available look‐back time was used to define the presence of diseases interrelated to obesity. The look‐back period in the United States was based on continuous time since enrollment, and in Japan it was based on time since first healthcare encounter.

### 2.3 Outcomes and Analysis

The following characteristics at index are reported: demographics (age and sex), BMI, and occurrence of diseases interrelated to obesity (dyslipidemia, hypertension, osteoarthritis, type 2 diabetes [T2D], atherosclerotic cardiovascular disease [ASCVD], heart failure [HF], chronic kidney disease [CKD], metabolic dysfunction–associated steatosis liver disease/metabolic dysfunction–associated steatohepatitis [MASLD/MASH], anxiety, depression, hypothyroidism, asthma, chronic obstructive pulmonary disease, and obstructive sleep apnea [OSA]; as defined based on diagnosis codes).

We described characteristics at index among those with a recorded diagnosis of obesity (International Classification of Diseases [ICD]‐9 278.0 or ICD‐10 E66) at any time (before or at index) versus those without such a diagnosis. For those with a recorded obesity diagnosis, we also described characteristics at the time of their first recorded obesity diagnosis in those first diagnosed during 2016–2020 (defined as “past diagnosis”) versus those first diagnosed in 2024 (defined as “contemporary diagnosis”). These time points were selected to consider the approximate timing of approvals of GLP‐1RAs for weight management in both countries. As such, data for those diagnosed between 2021 and 2023 are not shown as part of this analysis.

Differences in the occurrence of diseases interrelated to obesity between groups (with vs. without an obesity diagnosis and a past vs. contemporary diagnosis) were assessed by standardized mean differences (to allow the interpretation of differences on a uniform scale) with 95% confidence intervals (CIs). Standardized mean differences with 95% CIs that did not cross 0 were considered statistically significant.

## 3 Results

### 3.1 Study Sample

Overall, 3,584,120 (US dataset) and 3,140,900 (Japan dataset) participants met the country‐specific BMI criteria and had at least a 1‐year look‐back period before the index date. Among eligible individuals in the US dataset with BMI ≥ 30 kg/m^2^, 2,512,631 (70.1%) had a recorded obesity diagnosis and 1,071,489 (29.9%) did not. For individuals in the Japan dataset meeting the BMI ≥ 25 kg/m^2^ criteria, 72,609 (2.3%) had a recorded obesity diagnosis and 3,068,291 (97.7%) did not. Participant characteristics at index by country are summarized in Table 1.

**TABLE 1:** Demographics and clinical characteristics in participants with BMI ≥ 30 mg/kg^2^ (United States) or ≥ 25 mg/kg^2^ (Japan), with and without a recorded diagnosis of obesity.

	United States, BMI ≥ 30 kg/m^2^			Japan, BMI ≥ 25 kg/m^2^		
	Overall (*N* = 3,584,120)	With obesity Dx (*n* = 2,512,631)	Without obesity Dx (*n* = 1,071,489)	Overall (*N* = 3,140,900)	With obesity Dx (*n* = 72,609)	Without obesity Dx (*n* = 3,068,291)
Proportion of total *N*, %	100	70.1	29.9	100	2.3	97.7
Age, median (IQR), years	69.5 (59.3, 75.8)	69.9 (60.2, 76.1)	68.9 (56.1, 75.4)	67.0 (51.0, 77.0)	53.0 (40.0, 67.0)	67.0 (52.0, 77.0)
Female, *n* (%)	2,055,237 (57.3)	1,490,295 (59.3)	564,942 (52.7)	1,427,951 (45.5)	43,409 (59.8)	1,384,542 (45.1)
BMI, median (IQR), kg/m^2^	33.5 (31.1, 37.6)	34.3 (31.5, 38.7)	32.0 (30.7, 34.7)	27.1 (25.9, 29.1)	34.2 (30.1, 37.6)	27.0 (25.8, 29.0)

Abbreviation: Dx, diagnosis.

### 3.2 Burden of Diseases Interrelated to Obesity

The prevalence of diseases interrelated to obesity in the United States and Japan was high among individuals with elevated BMI; overall, 95.8% of participants with BMI ≥ 30 kg/m^2^ in the United States and 67.4% of participants with BMI ≥ 25 kg/m^2^ in Japan had at least one interrelated disease. In the United States, the prevalence of dyslipidemia was 80.4%, hypertension was 78.8%, T2D was 43.5%, OSA was 32.0%, CKD was 28.7%, and HF was 19.2%. In Japan, the prevalence of dyslipidemia was 30.8%, hypertension was 46.2%, T2D was 26.3%, OSA was 3.0%, CKD was 8.8%, and HF was 18.5%.

#### 3.2.1 With Versus Without an Obesity Diagnosis

Characteristics of participants at index by the presence versus absence of a recorded obesity diagnosis are summarized in Table 1. In both countries, there was a higher proportion of females among those with versus without an obesity diagnosis (United States: 59.3% vs. 52.7%; Japan: 59.8% vs. 45.1%). In Japan, participants with an obesity diagnosis were generally younger (53.0 vs. 67.0 years) than those without an obesity diagnosis. Median BMI was somewhat higher in those with versus without an obesity diagnosis in the United States (34.3 vs. 32.0 kg/m^2^) and higher in those with an obesity diagnosis in Japan (34.2 vs. 27.0 kg/m^2^).

**FIGURE 2:**
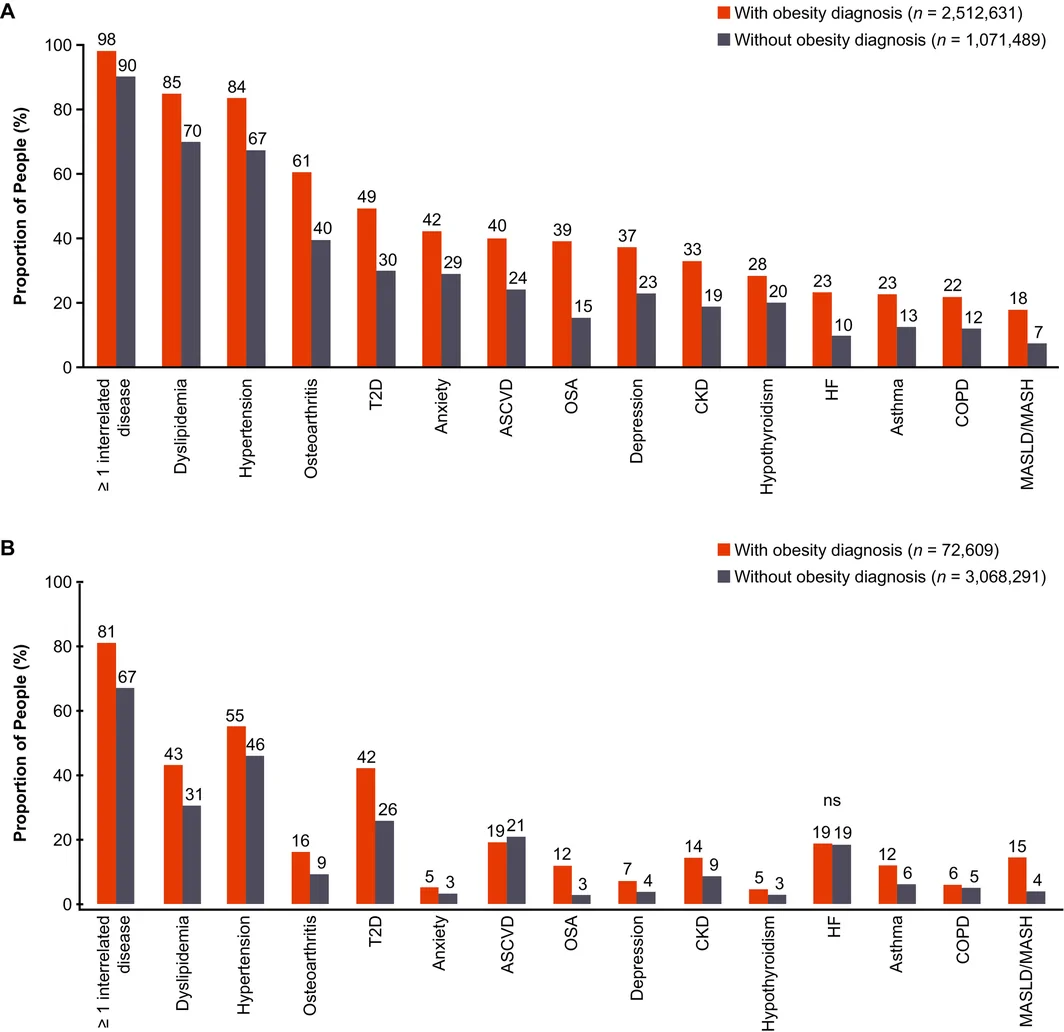
Prevalence of diseases interrelated to obesity in participants with elevated BMI, with versus without a recorded diagnosis of obesity, in (A) the United States (≥ 30 mg/kg^2^) and (B) Japan (≥ 25 mg/kg^2^). Standardized mean differences with 95% CIs that did not cross 0 were considered statistically significant. Differences were statistically significant for all interrelated diseases, except HF in Japan (denoted by “ns”). ASCVD, atherosclerotic cardiovascular disease; CKD, chronic kidney disease; COPD, chronic obstructive pulmonary disease; HF, heart failure; MASLD/MASH, metabolic dysfunction‐associated steatosis liver disease/metabolic dysfunction‐associated steatohepatitis; ns, not significant; OSA, obstructive sleep apnea; T2D, type 2 diabetes.

In both countries, the prevalence of diseases interrelated to obesity was generally higher in those with versus without an obesity diagnosis (Figure 2). In the United States, differences between those with versus without an obesity diagnosis were substantial for dyslipidemia (84.9% vs. 70.0%), hypertension (83.6% vs. 67.4%), osteoarthritis (60.5% vs. 39.5%), T2D (49.3% vs. 29.9%), OSA (39.1% vs. 15.4%), CKD (32.9% vs. 18.8%), and HF (23.3% vs. 9.8%). In Japan, differences between those with versus without an obesity diagnosis were notable for dyslipidemia (43.2% vs. 30.6%), T2D (42.2% vs. 25.9%), OSA (11.9% vs. 2.8%), CKD (14.3% vs. 8.6%), and MASLD/MASH (14.5% vs. 3.9%). Standardized mean differences in prevalences for those with versus without an obesity diagnosis are summarized in Table 2.

**TABLE 2:** Standardized differences in the prevalence of diseases interrelated to obesity in those with versus without a recorded obesity diagnosis.

Interrelated disease	United States, BMI ≥ 30 kg/m^2^		Japan, BMI ≥ 25 kg/m^2^	
	SMD	95% CI	SMD	95% CI
Dyslipidemia	0.36	0.36, 0.36	0.26	0.27, 0.26
Hypertension	0.39	0.39, 0.38	0.18	0.19, 0.18
Osteoarthritis	0.43	0.43, 0.43	0.21	0.21, 0.20
Type 2 diabetes	0.40	0.41, 0.40	0.35	0.36, 0.34
Anxiety	0.28	0.28, 0.28	0.10	0.10, 0.09
Atherosclerotic cardiovascular disease	0.35	0.35, 0.34	−0.04	−0.03, −0.05
OSA	0.55	0.56, 0.55	0.35	0.36, 0.35
Depression	0.32	0.32, 0.31	0.15	0.16, 0.14
Chronic kidney disease	0.33	0.33, 0.32	0.18	0.19, 0.17
Hypothyroidism	0.19	0.20, 0.19	0.09	0.10, 0.08
Heart failure	0.37	0.37, 0.37	0.01	0.02, 0.00
Asthma	0.27	0.27, 0.27	0.20	0.21, 0.19
Chronic obstructive pulmonary disease	0.27	0.27, 0.26	0.04	0.05, 0.03
MASLD/MASH	0.32	0.32, 0.31	0.38	0.38, 0.37

*Note:* SMDs with 95% CIs that did not cross 0 were considered statistically significant.

Abbreviations: MASLD/MASH, metabolic dysfunction‐associated steatosis liver disease/metabolic dysfunction‐associated steatohepatitis; OSA, obstructive sleep apnea; SMD, standardized mean difference.

#### 3.2.2 Contemporary Versus Past Obesity Diagnosis

Characteristics of participants at the time of the first recorded obesity diagnosis (contemporary diagnosis [in 2024] vs. past diagnosis [during 2016–2020]) are summarized in Table 3. In both countries, participants in the contemporary obesity diagnosis cohort were slightly younger than those diagnosed in the past (United States: 68.2 vs. 71.4 years; Japan: 52.0 vs. 54.0 years). In the United States, participants diagnosed more recently had a slightly lower median BMI than those diagnosed in the past (33.4 vs. 34.4 kg/m^2^); median BMI was more similar in the two cohorts in Japan (35.6 vs. 35.2 kg/m^2^).

**TABLE 3:** Demographics and clinical characteristics in participants with BMI ≥ 30 mg/kg^2^ (United States) or ≥ 25 mg/kg^2^ (Japan), by the time of their first recorded obesity diagnosis: past diagnosis (2016–2020) or contemporary diagnosis (in 2024).

	United States, BMI ≥ 30 kg/m^2^		Japan, BMI ≥ 25 kg/m^2^	
	Past obesity Dx (*n* = 1,265,566)	Contemporary obesity Dx (*n* = 213,798)	Past obesity Dx (*n* = 41,856)	Contemporary obesity Dx (*n* = 3295)
Age, median (IQR), years	71.4 (61.2, 77.3)	68.2 (55.4, 74.1)	54.0 (41.0, 68.0)	52.0 (40.0, 64.0)
Female, *n* (%)	758,020 (59.9)	122,865 (57.5)	25,095 (60.0)	1943 (59.0)
BMI, median (IQR), kg/m^2^	34.4 (31.5, 38.6)	33.4 (31.0, 37.0)	35.2 (31.2, 38.1)	35.6 (31.9, 38.3)

Abbreviation: Dx, diagnosis.

**FIGURE 3:**
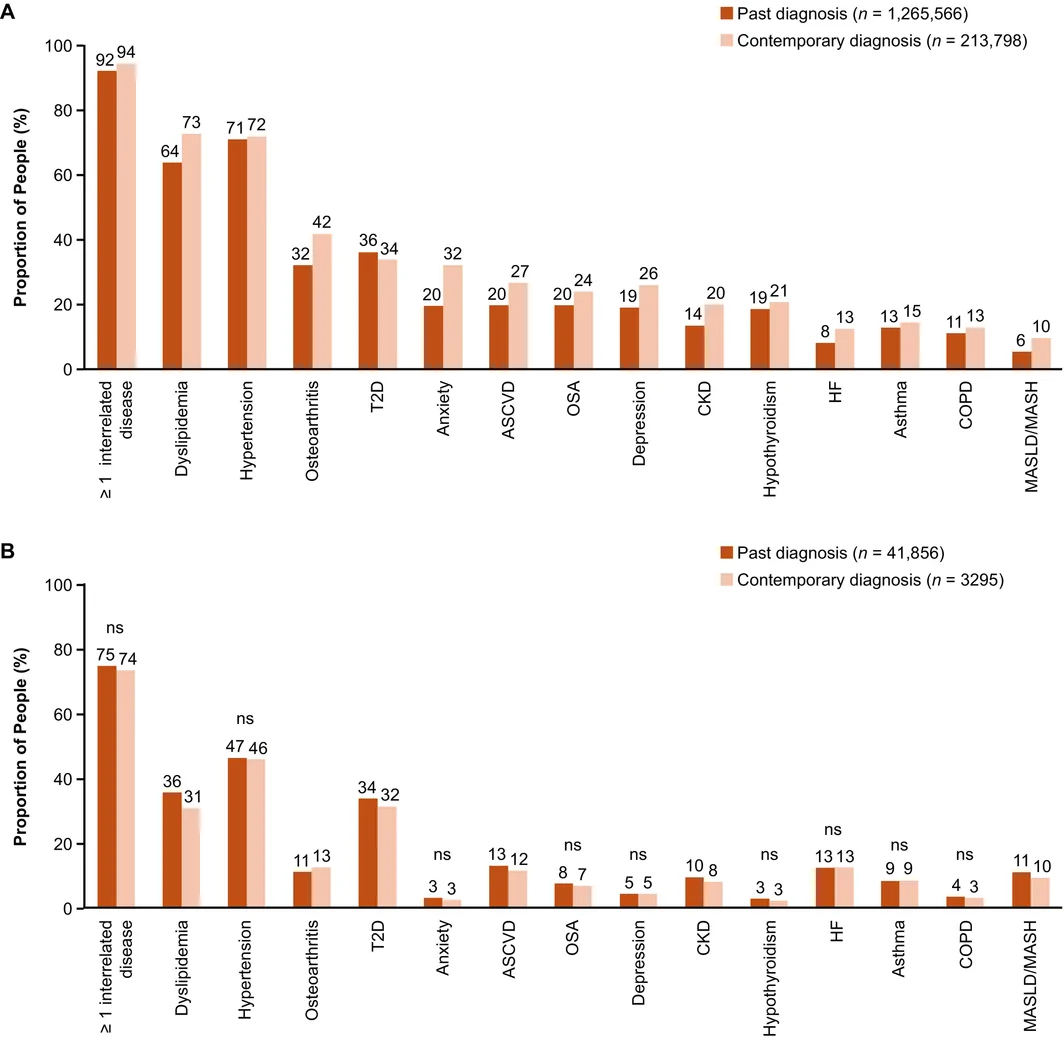
Prevalence of diseases interrelated to obesity in participants with elevated BMI, by the time of their first recorded obesity diagnosis (past diagnosis [2016–2020] or contemporary diagnosis [in 2024]), in (A) the United States (≥ 30 mg/kg^2^) and (B) Japan (≥ 25 mg/kg^2^). Standardized mean differences with 95% CIs that did not cross 0 were considered statistically significant. Differences that were not statistically significant are denoted by “ns.” ASCVD, atherosclerotic cardiovascular disease; CKD, chronic kidney disease; COPD, chronic obstructive pulmonary disease; HF, heart failure; MASLD/MASH, metabolic dysfunction‐associated steatosis liver disease/metabolic dysfunction‐associated steatohepatitis; ns, not significant; OSA, obstructive sleep apnea; T2D, type 2 diabetes.

In the United States, the burden of diseases interrelated to obesity was generally higher for those with a contemporary obesity diagnosis than those with a past obesity diagnosis (Figure 3A). Differences between those with a contemporary versus a past obesity diagnosis were most substantial for dyslipidemia (72.8% vs. 63.9%), osteoarthritis (41.9% vs. 32.2%), anxiety (32.2% vs. 19.6%), and CKD (20.0% vs. 13.5%). In Japan, the burden of diseases interrelated to obesity was generally similar or somewhat lower for those diagnosed more recently than the past obesity diagnosis cohort (Figure 3B). Differences between those with a contemporary versus a past obesity diagnosis were most substantial for dyslipidemia (31.1% vs. 35.9%), T2D (31.6% vs. 34.1%), CKD (8.3% vs. 9.6%), and MASLD/MASH (9.5% vs. 11.2%). Standardized mean differences in prevalences for those with a contemporary obesity diagnosis versus a past obesity diagnosis are summarized in Table 4.

**TABLE 4:** Standardized differences in the prevalence of diseases interrelated to obesity in those with a contemporary obesity diagnosis (in 2024) versus a past obesity diagnosis (during 2016–2020).

Interrelated disease	United States, BMI ≥ 30 kg/m^2^		Japan, BMI ≥ 25 kg/m^2^	
	SMD	95% CI	SMD	95% CI
Dyslipidemia	0.19	0.19, 0.20	−0.10	−0.14, −0.07
Hypertension	0.02	0.02, 0.03	−0.01	−0.04, 0.03
Osteoarthritis	0.20	0.20, 0.21	0.04	0.01, 0.08
Type 2 diabetes	−0.04	−0.05, −0.04	−0.05	−0.09, −0.02
Anxiety	0.29	0.28, 0.29	−0.03	−0.07, 0.00
Atherosclerotic cardiovascular disease	0.16	0.16, 0.17	−0.04	−0.08, −0.01
OSA	0.10	0.10, 0.11	−0.03	−0.07, 0.01
Depression	0.17	0.16, 0.17	0.00	−0.04, 0.04
Chronic kidney disease	0.18	0.17, 0.18	−0.05	−0.08, −0.01
Hypothyroidism	0.05	0.05, 0.06	−0.04	−0.07, 0.00
Heart failure	0.14	0.14, 0.15	0.00	−0.03, 0.04
Asthma	0.04	0.04, 0.05	0.00	−0.03, 0.04
Chronic obstructive pulmonary disease	0.06	0.05, 0.06	−0.01	−0.05, 0.02
MASLD/MASH	0.16	0.16, 0.16	−0.06	−0.09, −0.02

*Note:* SMDs with 95% CIs that did not cross 0 were considered statistically significant.

Abbreviations: MASLD/MASH, metabolic dysfunction‐associated steatosis liver disease/metabolic dysfunction‐associated steatohepatitis; OSA, obstructive sleep apnea; SMD, standardized mean difference.

## 4 Discussion

The findings from the present analysis of the multicountry, observational CALOR study showed underdiagnosis of obesity in people with elevated BMI ≥ 30 kg/m^2^ (United States) or ≥ 25 kg/m^2^ (Japan) in a contemporary real‐world setting. They also show a high prevalence of diseases interrelated to obesity, which was higher in those with versus without a recorded obesity diagnosis, as well as differences in diagnosis trends over time.

Thresholds for defining obesity by BMI in each country reflected local cutoffs (≥ 30 kg/m^2^ in the United States and ≥ 25 kg/m^2^ in Japan) [24]. However, it was considerably more common for participants with an elevated BMI to have a recorded obesity diagnosis in the United States (70.1%) than in Japan (2.3%). These differences between countries may reflect country‐specific sociodemographic and lifestyle differences related to obesity [26], ethnic and genetic differences in obesity pathophysiology [27], and differences in the data sources used (i.e., inpatient and outpatient data from commercially insured people in the United States vs. data from hospital care in Japan regardless of insurance). These findings may also reflect country‐specific differences in attitudes toward obesity and its management among people with obesity and healthcare providers (HCPs). For example, compared with other regions included in the ACTION‐IO survey (2018), lower proportions of people with obesity in Japan had discussed weight with their HCPs and greater proportions reported that they would not like their HCPs to discuss weight. Additionally, a lower proportion of HCPs felt comfortable during weight management conversations [28].

Previous studies have shown that undiagnosed obesity is common in clinical practice [4, 5, 6, 7, 8, 9, 10, 29]. Analyses of electronic medical records in the United States between 2004 and 2019 estimated that between 52% and 80% of people with BMI ≥ 30 kg/m^2^ did not have a formal diagnosis of obesity [4, 5, 6, 8, 9]. In a cross‐sectional study in the United States, significant underdiagnosis of obesity occurred among all types of HCPs [30]. Among patients with obesity as determined by BMI, non‐diagnosis rates were 54% for physicians, 67% for nurse practitioners, and 83% for residents [30]. In the IMPACT‐O cohort study (2018–2022), 94.2% of individuals with BMI ≥ 25 kg/m^2^ in the Japan cohort did not have an obesity diagnosis code in their medical record [29]. The findings of undiagnosed obesity in the present analysis (United States, 29.9%; Japan, 97.7%), observed in a more contemporary setting (up to and including 2024) and across diverse geographies, confirm those from previous studies and suggest no apparent improvement over time. Underdiagnosis of obesity is a major healthcare barrier, and improving diagnosis rates is a significant clinical opportunity, as recording an obesity diagnosis is associated with improved access to obesity healthcare and assessment of diseases interrelated to obesity [7]. In an observational study of 15 healthcare systems in the United States, a diagnosis of obesity was independently predictive of weight loss of ≥ 5% [5].

There are several potential reasons for the low diagnosis rates of obesity, including lack of recognition of obesity as a disease, social stigma, and lack of infrastructure to manage people with obesity (e.g., specialized facility equipment, resources, or training) [5, 8, 28]. Many countries do not recognize obesity as a disease, which has implications on treatment provision [31]. Furthermore, in the ACTION‐IO survey, only 58% of people living with obesity in Japan perceived obesity as a chronic disease [28]. People with higher BMI are more likely to have a recorded obesity diagnosis [30], suggesting that HCPs wait until the individual's condition is more severe before recording the diagnosis [6]. This was evident in our study in particular, where the median BMI of 34.2 kg/m^2^ for participants in Japan with an obesity diagnosis was almost 10 kg/m^2^ greater than the local BMI cutoff for obesity (≥ 25 kg/m^2^) [24]. There are also a number of barriers to conversations between HCPs and patients about obesity and how to manage this disease. In the ACTION‐IO survey in Japan, only 24% of people with obesity reported having weight management discussions with HCPs in the past 5 years; of those who had discussed weight, there had been a mean delay of 6 years between starting to struggle with their weight and their first conversation with an HCP [28]. Furthermore, in the same survey, almost half of HCPs reported that the responsibility to lose weight rested solely on the individual with obesity [28], indicating clear stigma and lack of understanding of obesity as a disease.

There are several proposed strategies for improving obesity diagnosis rates. Numerous leading institutions have recently sought to redefine obesity, aiming to promote measures with increased focus on adiposity (waist circumference, waist to height ratio, and percent body fat) and proposed combinations of these measures, to identify treatment targets that reduce the risk of interrelated diseases of obesity [32, 33]. US guidelines recommend screening all adults for obesity [11]. A missed obesity diagnosis is common in primary care, so approaches such as automated electronic medical record dashboard notifications for when a BMI threshold is recorded could improve rates of obesity diagnosis and increase the involvement of primary care providers in obesity care [7].

Obesity is complex, multifactorial, and associated with diverse interrelated conditions [1, 34, 35]; therefore, effective management should treat obesity as well as interrelated diseases [14, 36]. In the present analysis, the prevalence of diseases interrelated to obesity was high for participants with elevated BMI. Similar to previous findings in the United States [35], in the CALOR study, dyslipidemia and hypertension were the most common interrelated diseases in participants with obesity in the United States and Japan, although there was also a substantial burden of other disorders including CVRM diseases (T2D, CKD, and HF), respiratory disorders (OSA, asthma, and chronic obstructive pulmonary disease), and mental health conditions (anxiety and depression). In both countries, the prevalence of diseases interrelated to obesity was higher in participants with versus without a recorded obesity diagnosis. This finding may indicate that those without an obesity diagnosis are not receiving optimal care that identifies interrelated diseases, or that obesity is not diagnosed until a more progressed disease state, when interrelated diseases have already developed. Most participants with elevated BMI, including those without a recorded obesity diagnosis, had at least one disease interrelated to obesity at index. This finding is at variance with the concepts of “metabolically healthy obesity” and “preclinical obesity” (a term used in some frameworks), which are characterized by a state of excess adiposity but a lower risk of interrelated diseases, although the risk is still higher than in healthy, lean individuals [13, 37]. Understanding the range and prevalence of diseases interrelated to obesity may encourage HCPs and healthcare systems to systematically diagnose and better manage obesity and these interrelated diseases in individuals with elevated BMI, as well as to understand the risk of diseases interrelated to obesity in individuals who do not have them.

The higher and more complex interrelated disease burden in participants diagnosed more recently (2024) in the United States suggests a trend toward diagnosing obesity in a more progressed disease state. It is possible that the evolution of obesity treatment options, particularly the approval of GLP‐1RAs for this indication, has increased awareness of obesity and interrelated diseases among people with elevated BMI in the United States and encouraged them to present to their HCPs for diagnosis and treatment, particularly as individuals with either BMI ≥ 30 kg/m^2^ or ≥ 27 kg/m^2^ in the presence of comorbidities are candidates for GLP‐1RAs [15]. This observation of a higher and more complex interrelated disease burden may also reflect insurance providers setting additional clinical eligibility requirements than BMI ≥ 30 kg/m^2^ for their coverage of GLP‐1RAs, for example, a higher BMI cutoff plus at least one interrelated disease and therefore higher likelihood of interrelated disease identification and documentation [38, 39].

In Japan, the burden of diseases interrelated to obesity was similar or somewhat lower in the contemporary diagnosis cohort compared with the past diagnosis cohort. This may reflect a shift to an earlier diagnosis of the disease, before many interrelated diseases have developed, although this is uncertain as the rate of diagnosis in Japan was very low. The GLP‐1RAs semaglutide and tirzepatide were not approved in Japan until November 2023 and December 2024, respectively, which may not have permitted adequate time for similar findings as those observed in the United States to become visible, and the potential drivers for these country‐specific trends require further research.

Strengths of this study include the use of longitudinal data from healthcare claims and electronic medical records, reflecting routine clinical practice. The CALOR study assessed a large population across two countries (> 3 million in each) with different demographics, ethnicity‐related patterns of pathophysiology, and healthcare systems, therefore representing geographically diverse populations. Recommended region‐specific BMI thresholds were used, with the lower threshold for the Japanese population reflecting that Japanese individuals tend to develop related health disorders at a lower BMI than non–East Asian individuals [24].

There are also some limitations of the current analyses. As a surrogate marker, BMI thresholds can both underestimate and overestimate adiposity [40, 41]. There are likely individuals with obesity and excess adiposity who did not meet the BMI thresholds and were therefore excluded from the analysis; conversely, some individuals with elevated BMI without excess adiposity may have been included. Although BMI is not universally accepted to define obesity [13], it is widely used in routine clinical practice [12, 41] and so has advantages as a measure of obesity in epidemiological studies [13, 41]. The analyses were descriptive, not adjusted for confounders, and the findings of the interrelated diseases do not imply any temporal causality for, or effect from, obesity. Inherent to database studies, data are not originally collected for research purposes, and the validity in coding of diagnoses may be a limitation. The propensity to record BMI or a diagnosis code for obesity may be related to specific participant or provider characteristics, which may lead to selection bias. Clinical practice guidelines may vary across countries and clinical characteristics may be captured differently in the different data sources (i.e., the US data were from a commercially insured inpatient/outpatient population, compared with a hospital care setting irrespective of insurance status for Japan), potentially introducing bias. Use of obesity medications was not analyzed using the present data sources, as the capture of claims data in the United States likely underestimates the use of GLP‐1RAs due to the varying coverage across plans and due to the timings of regulatory approvals in Japan.

In conclusion, in people with elevated BMI across the United States and Japan, our data suggest that underdiagnosis of obesity in contemporary practice persists, representing a major barrier to obesity healthcare and assessment of interrelated diseases. The findings on the substantial burden of diseases interrelated to obesity among individuals with versus without a recorded obesity diagnosis, and over time in parallel with evolving obesity treatment options, may encourage HCPs and healthcare systems to systematically diagnose and better manage obesity and these interrelated conditions.

## Author Contributions

Conceptualization: H.W., M.B., D.B.H., E.L., and C.M.G. Methodology: E.L., M.B., and C.M.G. Software: C.M.G. Validation: All authors. Formal analysis: C.M.G. Investigation: C.M.G. and E.L. Resources: C.M.G. and E.L. Data curation: C.M.G. Writing – original draft: E.L. Writing – review and editing: H.W., M.B., D.B.H., E.L., and C.M.G. Visualization: H.W., M.B., D.B.H., E.L., and C.M.G. Supervision: H.W., M.B., D.B.H., E.L., and C.M.G. Project administration: E.L. Funding acquisition: E.L. and M.B.

## Funding

This study was sponsored by AstraZeneca.

## Conflicts of Interest

Prof. Horn has received consulting fees from Amgen, AstraZeneca, Boehringer Ingelheim, Eli Lilly, Novo Nordisk, and Zealand and institutional research funding from Eli Lilly, KVK Tech, Novo Nordisk, and Weight Watchers. Prof. Waki has received consulting fees from AstraZeneca AB and Novo Nordisk Health Care AG and honoraria for lectures from AstraZeneca K.K., Eli Lilly Japan K.K., Mitsubishi Tanabe Pharma Corporation, Novo Nordisk Pharma Ltd., Sumitomo Pharma Co. Ltd., Sanofi K.K., and Nippon Boehringer Ingelheim Co. Ltd. Drs. Lesén, Gray, and Bjursell are employees of, and may hold stock in, AstraZeneca.

## Data Availability

Data underlying the findings described in this article may be obtained from the corresponding author upon reasonable request, in accordance with AstraZeneca's data sharing policy described at https://astrazenecagrouptrials.pharmacm.com/ST/Submission/Disclosure. However, restrictions apply to these data, which were used under license and/or specific approval for the current study and are not publicly available.
